# A Mediating Effect of Psychological Distress in the Relationship Between Performance Status and Health-Related Quality of Life of Patients with Female Cancer

**DOI:** 10.3390/healthcare13091010

**Published:** 2025-04-28

**Authors:** Eunha Yeo, JinShil Kim, Jisun Yang, Eun Young Park, Kwang-Hi Park, KyungAh Cho, Seongkum Heo

**Affiliations:** 1 Gachon University Gil Medical Center, Incheon 21565, Republic of Korea; yeoeh@gilhospital.com; 2 College of Nursing, Gachon University, Incheon 21936, Republic of Korea; parkeunyoung@gachon.ac.kr (E.Y.P.); parkkh@gachon.ac.kr (K.-H.P.); jobari@gachon.ac.kr (K.C.); 3 Georgia Baptist College of Nursing, Mercer University, Atlanta, GA 30341, USA; heo_s@mercer.edu

**Keywords:** female, neoplasms, quality of life, functional status, psychological distress

## Abstract

**Objective:** This study aimed to examine whether anxiety and depressive symptoms mediate the relationship between performance status and health-related quality of life (HRQL) in patients with female cancer. A poor performance status is known to adversely affect HRQL and psychological distress—particularly anxiety and depressive symptoms—which may play a key role in this relationship. Identifying the mediating role of these symptoms may offer valuable insights into the mechanisms linking the performance status and HRQL. **Methods**: In a cross-sectional, correlational study, data on the HRQL (EORTC QLQ Version 3), performance status (Eastern Cooperative Oncology Group), and anxiety and depressive symptoms (Hospital Anxiety Depression Scale) were collected between February 2019 and June 2021. Process v4.1 for SPSS was used to analyze the data. **Results**: Sixty-five patients with female cancer participated (breast cancer = 44; gynecologic cancer = 21; mean age = 55.03 ± 8.65 years). Anxiety (*p* = 0.002), but not depressive symptoms (*p* = 0.525), mediated the relationship between the performance status and HRQL, explaining 41% of the variance in the HRQL (*R*^2^ = 0.41, *F* = 14.06, *p* < 0.001). A better performance status was only indirectly associated with a better HROL through the effect on anxiety. The total effect of the performance status on the HRQL was 15.972 (confidence interval [CI] = 6.095, 25.849): direct effect = 7.226 (CI = −1.936, 16.389) and indirect effect = 6.878 (standardized indirect effect = 0.374) (CI = 1.195, 15.395). **Conclusions**: The findings of this study only supported the mediating role of anxiety in the relationship between the performance status and HRQL in patients with female cancer. To improve the HRQL in patients with female cancer, improvements in the performance status and reductions in anxiety are critical.

## 1. Introduction

Despite diagnostic and therapeutic advances in cancer and prolonged survival [1], cancer remains a leading cause of death globally, accounting for an estimated 9.7 million deaths in 2022 and approximately 1 in 9 deaths of men and 1 in 12 deaths of women worldwide [2]. Cancers affecting the breast, lung, liver, ovary, cervix, uterine corpus, and colorectum contribute to approximately 60% of cancer burden in females [3]. Among them, cancers affecting the breasts and gynecologic organs are the most common among females [1,4] and are responsible for 14% of total deaths in females [5]. Among Korean adults, breast cancer is one of the most common cancers in women. In 2022, a total of 29,528 cases were reported, with an age-standardized incidence rate of 56.5 per 100,000 population [6]. Breast and gynecologic cancers accounted for approximately 13.7% of the total deaths in females [7].

Female cancer also can adversely impact health-related quality of life (HRQL). Cancer treatment and the relevant complications and side effects, such as changes in the self-perception of body image, cognitive function, and fatigue, considerably deteriorate HRQL in patients with female cancer [8,9]. According to a meta-analysis and systematic review [10], HRQL measured by the European Organization for Research and Treatment of Cancer Quality of Life C30) was relatively poor (pooled mean = 58.34 out of 100) in Asian patients with breast cancer. Furthermore, poor HRQL in patients with female cancer adversely affects the mortality rates [11]. Enhancing HRQL among patients with female cancer is thus a clinical and research priority.

To improve HRQL in patients with female cancer, it is essential to identify modifiable factors associated with HRQL. One such factor is performance status, which refers to a patient’s ability to perform activities of daily living [12,13]. Performance status is often impaired in this population due to cancer treatment and its side effects [14]. For example, patients with breast cancer frequently experience limited shoulder mobility, difficulty in walking, and challenges in performing routine household tasks [14,15]. Another critical factor influencing HRQL is psychological distress. Psychological distress is a broad term that encompasses emotional suffering, typically manifested as anxiety, depressive symptoms, or both [16,17]. It is highly prevalent among patients with female cancer due to the psychological burden of diagnosis, treatment, and concern about recurrence [18,19]. Previous studies have reported that approximately 37% to 47% of female cancer patients experience clinically significant levels of anxiety or depressive symptoms. These psychological symptoms have consistently been linked to reduced HRQL [18,19,20,21,22].

Supportive care needs, especially in the psychological domain, are central to improving the HRQL in patients with cancer but remain insufficiently addressed in clinical practice [23]. Addressing these needs is essential to improve patient outcomes. Importantly, performance status has also been associated with psychological distress, suggesting a possible pathway through which performance status may influence HRQL indirectly. For example, patients with a lower performance status tend to report higher levels of anxiety and depressive symptoms [18]. These findings point to the potential mediating role of psychological distress in the relationship between performance status and HRQL. That is, direct associations of performance status, anxiety, and depressive symptoms with HRQL, and the association between performance status and anxiety and depressive symptoms, imply that anxiety and depressive symptoms may mediate the relationship between performance status and HRQL. However, this mediating relationship has not been adequately examined, particularly among patients with female cancer. Although one study that involved a mixed gender sample (43.6% female patients with cancer) found some links between these variables, the results were inconsistent and limited to certain HRQL dimensions [24]. In that study, both performance status and depressive symptoms were independently associated with most HRQL dimensions, but a significant interaction was found only for global HRQL. Given the high prevalence of psychological distress in this population and its known impact on HRQL, further investigation is warranted. Specifically, it is important to examine where anxiety and depressive symptoms mediate the relationship between performance status and HRQL. Addressing this gap may help clarify the mechanisms linking physical and psychological health in female cancer patients and inform more effective, targeted interventions.

Understanding both the direct and indirect effects of performance status on HRQL through anxiety and depressive symptoms can be critical for improving the HRQL in patients with female cancer. Female patients with cancer may be especially vulnerable to psychological distress and poor HRQL compared with males or those with other types of cancer [25,26,27]. This vulnerability may stem from their caregiving roles, a lack of adequate support, and the negative consequences of cancer treatment [8,28]. For example, women are more likely to take on primary caregiving responsibilities, often managing the care of children, spouses, and elderly parents, while also attending to their own health needs [28]. These caregiving roles are deeply ingrained in Asian cultures and can lead to an additional psychological burden when women face illness. Female patients with breast cancer often report a lack of support, prompting them to seek external support networks [29]. Even in Asian women with gynecological cancer, intimate partner support is not always present [30]. Additionally, concerns about a loss of sexuality or femininity further contribute to psychological distress [30].

Taken together, these factors highlight the importance of examining the dynamic relationships between performance status, psychological symptoms, and HRQL in this population. Given the psychological vulnerability linked to caregiving roles and limited support, anxiety and depressive symptoms may serve as critical pathways linking performance status and HRQL. This study specifically focused on patients with female cancer who had completed both curative surgery and adjuvant chemotherapy, excluding those in palliative care. This approach was taken to reduce the clinical heterogeneity and focus on recovery-related issues rather than end-of-life care. Therefore, the aim of this study was to examine whether anxiety and depressive symptoms simultaneously mediate the relationship between the performance status and HRQL in patients with female cancer. We hypothesized that a poor performance status would be associated with higher levels of both anxiety and depressive symptoms, which, in turn, would be associated with a poorer HRQL.

## 2. Materials and Methods

### 2.1. Design and Setting

Using a cross-sectional, correlational study design, this study was undertaken to examine the mediating role of psychological symptoms in the relationship between the performance status and HRQL among patients with female cancer. Eligible patients were screened and enrolled from the outpatient clinic of a single university-affiliated hospital located in a metropolitan city in South Korea between February 2019 and June 2021. This hospital was selected based on its high patient volume, accessibility, and availability of specialized oncology services for female cancers. The institution also had an established research collaboration with the study team, which facilitated the participant recruitment and data collection.

### 2.2. Sample and Sampling Method

Patients with female cancer were recruited using a convenience sampling method. Eligibility criteria included (1) adults aged 19 years or older, (2) diagnosed with cancer in the breasts or gynecologic organs (ovaries, uterus, or cervix), and (3) having undergone surgery with curative intent followed by adjuvant chemotherapy. Exclusion criteria were (1) documented or current comorbid conditions of psychiatric disorders (e.g., affective disorders); (2) limited life-expectancy or receipt of hospice or palliative care; or (3) conditions that impaired vision, hearing, or comprehension of the study materials.

Using G-power 3.1.9.7 [31], a sample size for a mediating model test was computed with a two-tailed test. The mediation analysis was conducted using Process macro v4.1 for SPSS (Model 4) with bootstrap. Although the mediating roles in these specific relationships have rarely been examined in this population, previous studies have reported significant associations between performance status and psychological symptoms, between performance status and HRQL, and between psychological symptoms and HRQL [13,18,19]. Performance status was significantly associated with HRQL (*p* < 0.001), and psychological symptoms explained 49% of the variance in HRQL [13,19]. Furthermore, performance status was strongly associated with psychological symptoms (*p* = 0.002 and 0.001) in this population [18]. Based on these findings and in line with the guidelines by Sim and colleagues [32], the anticipated sample size for detecting partial or complete mediation, assuming a large effect size and bootstrap approach, was 50 for a multiple mediation model (Model 2) [32]. In this study, 65 patients with female cancer completed the survey questionnaires.

### 2.3. Data Collection

The first author collected data on the HRQL, performance status, anxiety, and depressive symptoms at outpatient visits for routine care through face-to-face interviews according to the study protocol that indicated detailed instructions about this study and the data collection process. If it was needed, the first author provided assistance to the research participants to complete the questionnaires. The average data collection time was 20–30 min.

### 2.4. Measurements

Quality of life: The summary score of the European Organization for Research and Treatment of Cancer Quality of Life C30 (EORTC QLQ-C30, Ver. 3.0) was used to assess the HRQL in this study [33]. The EORTC QLQ-C30 consists of 30 items with 15 subscales. The functional and symptom scales use 4-point Likert scales. For this study, the summary score of QLQ-C30, which included 13 of 15 subscales, but excluded the global health status/HRQL and the financial scales, was used to assess the HRQL. A 0-to-100 transformed score was computed based on the EORTC scoring guidelines [34], with a high score indicating a greater HRQL. The reliability and validity of the original [33] and Korean [35] versions were supported. In the present study, Cronbach’s alphas ranged from 0.88 to 0.89 for the functional scales, and from 0.86 to 0.87 for the symptom scales. The global health status/QoL scale showed an alpha of 0.91.

Psychological distress: Anxiety and depressive symptoms were assessed using the 14-item Hospital Anxiety and Depression Scale (HADS), which is evenly divided into anxiety and depression subscales [36]. Each item of the HADS is rated using a 4-point Likert scale, ranging from 0 to 3. The possible total score ranges from 0 to 21 for each subscale. Higher scores indicate more severe anxiety or depressive symptoms. The cut-off points of 8 or higher were used to determine the prevalence of anxiety and depressive symptoms [36]. The reliability and validity of the original [36] and Korean [37] versions were supported. In the present study, Cronbach’s alpha was 0.80 for both the anxiety and depression subscales.

Performance status: The performance status was assessed using the Eastern Cooperative Oncology Group (ECOG) performance scale [38]. This instrument is used to assess the effects of a therapeutic regiment on an individual’s performance of daily living activities [39]. The performance status is classified from 0 (fully active) to 4 (completely disabled). Higher numbers indicate a poorer performance status. In this study, the ECOG scores were dichotomized with the cut-off point of 2 (0–1 vs. 2–4), with scores of 0–1 indicating a normal performance status and scores of ≥2 indicating an impaired status. This approach is supported by previous studies showing that ECOG ≥ 2 predicts survival outcomes in patients with cancer [40] and demonstrates discriminative and predictive validity in lung cancer [41].

### 2.5. Statistical Analysis

Descriptive statistics were computed to describe the sample characteristics and study variables. In the Process macro v3.3 for SPSS [42], Model 4 (i.e., a simple mediator model) was adopted to examine the mediating effects of anxiety and depressive symptoms in the relationship between the performance status and HRQL. In the model, the performance status was used as the independent variable, the anxiety and depressive symptoms as the mediators, and the HRQL as the outcome variable. The significance level was set at *p* < 0.05, and two-tailed tests were applied to all the analyses.

## 3. Results

### 3.1. Characteristics and HRQL of Patients with Female Cancer

Sixty-five patients with female cancer participated in this study (Table 1). The mean age was 55.0 years (±standard deviation 8.6; range 31–79). The majority of the participants were married 84.6%, had a religious affiliation (63.1%), received a high school or higher education (73.8%), maintained their employment status (83.1%), and had family caregivers (75.4%). The types of female cancers were mostly breast cancer (67.7%) and gynecologic cancers (32.3%) in an ovary (6.2%), cervix (15.4%), uterus (6.2%), and vulva (4.6%). Nearly half of the patients had breast cancer stage I (40%), followed by II (30.8%), III (18.5%), and IV (4.6%). In this sample, 52.3% had anxiety (≥8 points) and 35.4% had depression (≥8 points).

### 3.2. The Results of the Performance Status, Psychological Distress, and Health-Related Quality of Life and the Relationships

Sixteen patients (24.6%) had a moderately poor performance status (ECOG score: 2–4) (Table 1). The score for the HRQL among the patients with female cancer was 67.32 ± 18.39 out of 100 (Table 2). The scores for anxiety and depressive symptoms were 8.14 ± 4.23 out of 21 and 6.82 ± 4.44 out of 21, respectively. In the bivariate analyses (Table 2), the performance status was significantly correlated with anxiety (r = 0.41), depressive symptoms (r = 0.49), and the HRQL (r = –0.54). Anxiety was significantly correlated with depressive symptoms (r = 0.79) and the HRQL (r = −0.61), and depressive symptoms were also correlated with the HRQL (r = −0.55) (all *ps* < 0.01). Specifically, a poorer performance status was associated with greater psychological distress (i.e., more severe anxiety and depressive symptoms) and a poorer HRQL.

### 3.3. Mediating Roles of Anxiety and Depressive Symptoms in the Relationship Between the Performance Status and HRQL

**Figure 1:**
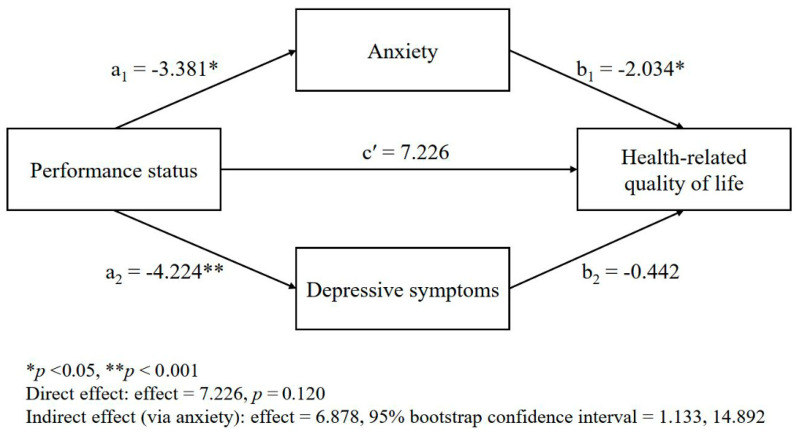
Multiple mediation model: mediating effects of psychological distress (anxiety and depressive symptoms) in the relationship between the performance status and the health-related quality of life.

The mediating roles of anxiety and depressive symptoms in the relationship between the performance status and HRQL are presented in Table 3 and Figure 1. The performance status was significantly associated with both anxiety (*b* = −3.38, *t* = −2.94, *p* = 0.005) and depressive symptoms (*b* = −4.22, *t* = −3.60, *p* < 0.001), indicating that a poorer performance status was related to greater psychological distress. However, in the final model that predicted the HRQL, only anxiety remained a significant predictor (*b=* −2.03, *t* = −2.89, *p* = 0.005), whereas depressive symptoms did not (*b =* −0.44, *t* = −0.64, *p* = 0.525). The direct effect of the performance status on the HRQL was no longer significant (*b =* 7.23, *t* = 1.58, *p* = 0.120). These findings suggest that anxiety, but not depressive symptoms, fully mediated the relationship between the performance status and HRQL (Figure 1). The indirect effect of the performance status on the HRQL through anxiety was statistically significant (indirect effect = 6.88; boot confidence interval = 1.13, 14.89), indicating that individuals with a poorer performance status experienced more severe anxiety, which, in turn, was associated with a poorer HRQL.

## 4. Discussion

The findings of this study partially supported the hypothesis that a poor performance status would be associated with anxiety and depressive symptoms, and, in turn, the HRQL. Only anxiety, but not depressive symptoms, mediated the relationship between the performance status and HRQL. The performance status of patients with female cancer was associated with the HRQL only indirectly through the effect on anxiety. These findings highlight the central role of anxiety as a psychological mechanism that linked the performance status and HRQL.

The overall findings in the literature regarding the direct relationships of the performance status, anxiety, and depressive symptoms with the HRQL [13,15,19,20,21,22] and the direct relationships of anxiety and depressive symptoms with the performance status [18] suggest the mediating roles of anxiety and depressive symptoms in these relationships. However, these relationships have been rarely examined in this population. The findings of this study emphasize the need for the simultaneous consideration of both performance status and anxiety to improve the HRQL in patients with female cancer. Miniotti et al. [43] also highlighted the importance of addressing both functional and psychological needs in oncology, emphasizing that supportive care should integrate psychological support, along with functional care, to optimize HRQL outcomes. This aligns with the results of the current study, where anxiety played a pivotal role in the relationship between the performance status and HRQL. Considering the mean scores of HRQL among general populations in Europe (84.3–86.2 out of 100) [44], the HRQL among female cancer survivors in this study was low (67). A meta-analysis of HRQL in patients with breast cancer found similar global HRQL scores (64.7) [45], and in Iranian patients with breast cancer, as well as a meta-analysis of Asian patients with breast cancer, the global or overall HRQL scores were even lower (58–59 out of 100) [10,46]. All of these findings emphasize the need for improved HRQL among patients with female cancers.

The findings of this study suggest two important intervention targets for an improvement in the HRQL among patients with female cancer. In this study, one modifiable intervention target to improve HRQL was the performance status, which is consistent with that in the literature [13]. Approximately one-fourth (24.6%) of patients with female cancer had a moderately poor performance status (ECOG score: 2–4), which aligns with findings from Miniotti et al. [43], where a loss of functioning significantly affected the HRQL in patients with advanced cancer. In previous studies [14,15], patients with breast cancer also had limited performance in their upper extremities, mobility, and household tasks, suggesting that a poor or limited performance status needs to be improved. However, in contrast to the direct relationship of performance status to HRQL in patients with female cancers [12,13,47], the performance status was not directly associated with the HRQL in this study. Among the previous studies that showed the direction relationship between performance status and HRQL, two did not include anxiety in their models [12,13], and one study [47] found a relationship in bivariate analyses, but it was not included in the multivariable analysis. In contrast, another study [48] did not find an association between performance status and HRQL in both univariate and multivariable analyses. Therefore, further studies are needed to examine the direct relationship between performance status and HRQL, considering other potential factors that can be associated with HRQL, such as symptom burden.

The performance status in this study was associated with the HRQL only through anxiety. A poor performance status was significantly associated with more severe anxiety, which, in turn, was related to a poor HRQL. The direct relationships between performance status and anxiety and between anxiety and HRQL were also supported in previous studies [18,20]. The findings imply that a poor performance status and anxiety should be improved simultaneously to improve HRQL in patients with female cancer. Furthermore, anxiety should be targeted because it is highly prevalent among patients with female cancer. In the literature [18,19], the prevalence of anxiety in patients with female cancer ranges from 44.2% to 47.2%, which is consistent with the prevalence in this study (52.5%). Therefore, interventions targeting both the performance status and anxiety are needed to improve the HRQL in this population.

A poor performance status was also associated with more severe depressive symptoms in this study, but depressive symptoms were not associated with the HRQL. Thus, depressive symptoms did not mediate the relationship in this sample. The mediator role of depressive symptoms in the relationship between performance status and HRQL among patients with female cancer has not been frequently examined. Nonetheless, the direct relationship between performance status and depressive symptoms was also supported in a previous study [18]. In contrast to the current findings, depressive symptoms were directly associated with HRQL in prior studies involving adult or breast cancer patients [12,48]. Notably, the breast cancer study [12] did not include anxiety in the model, whereas the study of adult cancer patients [48] did, although it encompassed both sexes and multiple cancer types. Dionish-Vici et al. also emphasized that psychological distress, particularly anxiety, tends to persist in cancer survivors, whereas depressive symptoms often show a limited influence on HRQL [49]. This may help explain the non-significant association found in the present study. As suggested by Dionish-Vici et al. [49], addressing unmet psychological needs, especially anxiety, should be prioritized in efforts to improve HRQL. Given the lower prevalence and severity of depressive symptoms relative to anxiety in this sample, the lack of association with HRQL may be partly attributed to this discrepancy. Building on this, psychological symptoms—particularly anxiety—should be a central focus of intervention strategies. In addition to psychological support, growing evidence supports the role of physical activity [50,51]. Clinicians may consider integrating appropriate exercise interventions, such as aerobic or resistance training, as part of a comprehensive care approach. These forms of exercise have been shown to enhance the performance status, reduce anxiety, and improve HRQL, offering a valuable adjunct to cancer care.

In this study, there were several limitations. First, the sample predominantly consisted of breast cancer patients, with only a small number of patients with ovarian cancer. Given the potential differences in performance status, psychological distress, and HRQL across cancer types, the findings may not be fully generalizable to all patients with female cancer. Second, the cancer stage was not considered in the mediation model, as it was not part of the variables considered in the a priori power analysis. Including the cancer stage may have provided additional insights into the relationships between the performance status, psychological distress, and HRQL. Third, although this study used a mediation model, its cross-sectional design limited the ability to infer causal or directional relationships between variables. A mediation analysis assumes a directional pathway, but the temporal ordering of variables cannot be established in this design. Furthermore, individuals with a limited life expectancy or those who had been diagnosed but had not yet undergone both surgery and chemotherapy were inherently excluded. These exclusions may further limit the generalizability of the findings to the broader population of patients with cancer. Last, the dichotomization of ECOG scores may have led to a loss of information, although this approach was based on established clinical cut-offs and prior studies.

Future studies are warranted with a similar composition of breast and other types of female cancer (ovarian cancer, cervix, and endometrium). In addition, future studies with appropriately powered sample sizes are needed to examine the mediator roles of anxiety and depressive symptoms while accounting for important covariates such as the cancer stage. Other potential covariates to consider include having children or being a caregiver [13,21,46], as well as the time after cancer diagnosis [21,46] in breast or gynecologic cancer.

## 5. Conclusions

In Korean patients with female cancer, a poor performance status was associated with a poor HRQL through anxiety, but not through depressive symptoms, partially supporting the hypothesis. The findings contribute to the growing body of evidence on the psychological mechanisms linking performance status and quality of life, highlighting anxiety as a key mediator. From an evidence-based clinical perspective, the results suggest two important targets of interventions to improve the HRQL in this population. First, enhancing the performance status may lead to reductions in anxiety, thereby improving the HRQL. Second, given the direct effect of anxiety on the HRQL, targeted psychological interventions aimed at reducing anxiety may also be effective. This mediation model provides a theoretical foundation for developing more holistic and integrated interventions that simultaneously address both physical and emotional well-being. The routine assessment and management of both performance status and anxiety are therefore recommended in clinical practice to help maintain or improve the HRQL in patients with female cancer.

## Figures and Tables

**Table 1:** Sample characteristics (N = 65).

Characteristics	Categories	Mean ± SD	n (%)
Age (years)		55.0 ± 8.6	
Marital status	Single		3 (4.6)
	Married		55 (84.6)
	Divorced		5 (7.7)
	Widow		2 (3.1)
Religious affiliation, yes	Yes		41 (63.1)
Educational levels	<High school		17 (26.2)
	=High school		26 (40.0)
	≥College		22 (33.8)
Employment, yes	Employed		23 (35.4)
Family caregivers, yes	Yes		49 (75.4)
Types of cancer	Breast		44 (67.7)
	Cervix		10 (15.4)
	Ovary		4 (6.2)
	Endometrium		4 (6.2)
	Vulva		3 (4.6)
Stages of cancer	Stage 0		4 (6.2)
	Stage I		26 (40.0)
	Stage II		20 (30.8)
	Stage III		12 (18.5)
	Stage IV		3 (4.6)
Time after cancer diagnosis (years)	<1.00		37 (56.9)
	1.00–2.99		10 (15.4)
	≥3		18 (27.7)
ECOG	0		17 (26.2)
	1		32 (49.2)
	2		7 (10.8)
	3		7 (10.8)
	4		2 (3.1)

Abbreviations: ECOG, Eastern Cooperative Oncology Group; SD, standard deviation.

**Table 2:** Bivariate relationships between functional status, anxiety, depressive symptom, and health-related quality of life (N = 65).

Variables	Mean ± SD	Pearson’s Correlation Coefficient			
		Performance Status	Anxiety	Depressive Symptoms	HRQL
Performance status	1.15 ± 1.03	1			
Anxiety	8.14 ± 4.23	0.41 *	1		
Depressive symptoms	6.82 ± 4.44	0.49 *	0.79 *	1	
HRQL	67.32 ± 18.39	−0.54 *	−0.61 *	−0.55 *	1

Abbreviations: SD, standard deviation; HRQL, health-related quality of life. Potential score ranges: performance status: 0–4; anxiety: 0–21; depressive symptoms: 0–21; and HRQL: 0–100. * *p* < 0.001.

**Table 3:** The mediating effects of psychological distress (anxiety, depressive symptom) in the relationship between the performance status and health-related quality of life (N = 65).

**Path Coefficients from Mediation Model**								
Variable Relationships		b	β	SE	*t*	*p*	LLCI	ULCI
Performance status → Anxiety		−3.381	−0.800	1.151	−2.94	0.005	−5.681	−1.082
Performance status → Depressive symptoms		−4.224	−0.952	1.172	−3.60	<0.001	−6.567	−1.882
Anxiety → HRQL		−2.034	−0.468	0.704	−2.89	0.005	−3.442	−0.626
Depressive symptoms → HRQL		−0.442	−0.107	0.691	−0.64	0.525	−1.824	0.940
Performance status → HRQL		7.226	0.393	4.582	1.58	0.120	−1.936	16.389
**Indirect Effects of Performance Status**								
Mediator		b	β	Boot SE	Boot LLCI		Boot ULCI	
Anxiety		6.878	0.374	3.575	1.133		14.892	
Depressive symptoms		1.868	0.102	3.217	−3.829		9.206	
**Total and Direct Effects of Performance Status on HRQL**								
Effect type	b	β	SE	*t*	*p*		LLCI	ULCI
Total	15.972	0.869	4.943	3.23	0.002		6.095	25.849
Direct	7.226	0.393	4.582	1.58	0.120		−1.936	16.389

Abbreviations: ECOG—Eastern Cooperative Oncology Group; HRQL—health-related quality of life; Boot SE—bootstrapped standard error, an estimate of the standard error obtained through resampling methods; LLCI—lower level of the 95% confidence interval based on bootstrap estimates; SE—standard error; ULCI—upper level of the 95% confidence interval based on bootstrap estimates. Note: performance status was dichotomized with ECOG scores of 0, 1 vs. 2–4.

## Data Availability

Dataset available upon request from the authors.
